# Two eggs, two different constraints: a potential explanation for the puzzling intraclutch egg size dimorphism in *Eudyptes* penguins

**DOI:** 10.1002/ece3.1543

**Published:** 2015-06-25

**Authors:** Maud Poisbleau, Nina Dehnhard, Laurent Demongin, Petra Quillfeldt, Marcel Eens

**Affiliations:** 1 Department of Biology – Ethology, University of AntwerpCampus Drie Eiken, Universiteitsplein 1, 2610, Antwerp, Wilrijk, Belgium; 2 Max Planck Institute for Ornithology, Department of Migration and Immuno-Ecology, Am Obstberg 178315, Radolfzell, Germany; 3 Department of Biology, University of Konstanz78457, Konstanz, Germany; 4 Department of Animal Ecology & Systematics, Justus-Liebig University GießenHeinrich-Buff-Ring 38, 35392, Gießen, Germany

**Keywords:** Body reserves, constraints, egg composition, maternal investment

## Abstract

Phenotypic plasticity and phenotypic stability are major components of the adaptive evolution of organisms to environmental variation. The invariant two-egg clutch size of *Eudyptes* penguins has recently been proposed to be a unique example of a maladaptive phenotypic stability, while their egg mass is a plastic trait. We tested whether this phenotypic plasticity during reproduction might result from constraints imposed by migration (migratory carry-over effect) and breeding (due to the depletion of female body reserves). For the first time, we examined whether these constraints differ between eggs within clutches and between egg components (yolk and albumen). The interval between colony return and clutch initiation positively influenced the yolk mass, the albumen mass, and the subsequent total egg mass of first-laid eggs. This time interval had only a slight negative influence on the yolk mass of second-laid eggs and no influence on their albumen and subsequent total masses. For both eggs, female body mass at laying positively influenced albumen and total egg masses. Female investment into the entire clutch was not related to the time in the colony before laying but increased with female body mass. These novel results suggest that the unique intraclutch egg size dimorphism exhibited in *Eudyptes* penguins, with first-laid eggs being consistently smaller than second-laid eggs, might be due to a combination of constraints: a migratory carry-over effect on the first-laid egg and a body reserve depletion effect on the second-laid egg. Both these constraints might explain why the timing of reproduction, especially egg formation, is narrow in migratory capital breeders.

## Introduction

Phenotypic plasticity is the ability of an individual organism (single genotype) to express different phenotypes (morphology, physiology, behavior, and/or life history) in different environments (West-Eberhard 1989; Scheiner 1993). In contrast, phenotypic stability (i.e., canalization against environmental variation) describes a reduction in phenotypic plasticity in response to environmental variation. Canalized traits tend to produce invariant phenotypes under a wide range of environmental conditions (Stearns and Kawecki 1994; Debat and David 2001). Traits that are directly related to fitness (reproduction and survival) should tend to be more canalized to allow organisms to maintain high fitness levels across environments, while traits that are not directly related to fitness should tend to be more plastic, especially when environmental variation is expected (Liefting et al. 2009). Both phenotypic plasticity and phenotypic stability may therefore be observed in response to extrinsic (biotic or abiotic) constraints and play a role in the process of adaptive evolution in new environments (Crespi 2000; DeWitt and Scheiner 2004; Ghalambor et al. 2007; Lande 2009; Le Rouzic et al. 2013). However, although information on their proximate and ultimate causes is crucial to obtain a comprehensive understanding of the evolution of life-history traits and strategies (Stearns 1977; Roff 2002), few studies have been conducted under natural conditions.

Clutch size of *Eudyptes* penguins has recently been proposed to be a unique example of maladaptive phenotypic stability (Stein and Williams 2013). *Eudyptes* penguins lay an invariant two-egg clutch, but the first-laid A-egg, which is always smaller than the second-laid B-egg (A-egg/B-egg volume ratios range from 0.61 to 0.79, Demongin et al. 2010), almost never produces a fledging chick (Lamey 1990; Poisbleau et al. 2008). This results in an A-egg with no apparent adaptive function, and an evolutionary mismatch between clutch size and realized fecundity (number of chicks fledged per two-egg clutch), which has puzzled evolutionary biologists for decades (Gwynn 1953; Lack 1968; Williams 1980, 1990; Johnson et al. 1987; St. Clair 1992, 1995, 1998; Demongin et al. 2010). A recent comparison among penguin species (Stein and Williams 2013) showed that the seven *Eudyptes* species show a slower pace of life, with a later age of first reproduction and lower annual fecundity than the other penguin genera that produce two-egg clutches. Moreover, other bird species that show a similar pace of life (e.g., relative egg size and annual fecundity) produce only one-egg clutches. It therefore appears that *Eudyptes* penguins failed to evolve a one-egg clutch despite a marked life-history slowdown (Stein and Williams 2013). This clutch size maladaptation is associated with and may partially explain the unique extreme intraclutch egg size dimorphism observed in *Eudyptes* penguins.

In contrast to clutch size, egg size is a plastic trait in *Eudyptes* species. Egg size and the related intraclutch egg size dimorphism show large variation among *Eudyptes* species, populations, and individuals as well as between breeding events for individual females (Warham 1975; Demongin et al. 2010). Accordingly, Crossin et al. (2010) hypothesized that the extreme intraclutch egg size dimorphism observed in *Eudyptes* penguins was due to a physiological constraint imposed by a migratory carry-over effect. These authors showed that the degree of intraclutch egg size dimorphism was inversely correlated with the time interval between colony return and clutch initiation (or, conversely, the variation in the amount of time spent migrating while producing eggs). Females that laid shortly after their return to the colony showed stronger migratory carry-over effects with lower reproductive readiness (as indicated by plasma yolk precursor levels) and more dimorphic clutches than females laying later after their return (Crossin et al. 2010). To improve our understanding of the proximate causes of this migratory carry-over effect, it is necessary to test how the conflict between migration and reproduction acts on the different eggs and their components (especially yolk and albumen). Variation in egg composition is likely an important component determining intraclutch egg size dimorphism in penguins. Previous investigations suggested that the proportion of albumen increased, while the proportion of yolk decreased relative to total egg mass as egg mass increased, resulting in a lower proportion of albumen and a higher proportion of yolk in A-eggs compared with the B-eggs (Williams et al. 1982; Gwynn 1993; St. Clair 1996).

In *Eudyptes* penguins, yolk production lasts around 16 days (Grau 1982; Crossin et al. 2010). The yolk is thereafter retained within the ovarian follicle for 6 days before the albumen and shell are added during the last day(s) before ovulation (Grau 1982). Egg production therefore takes 23 days in total (Grau 1982). In southern rockhopper penguins *Eudyptes chrysocome*, our study species, females return from their winter migration to the colony about 10 days before clutch initiation (A-egg laying) (Strange 1982; Ancel et al. 2013). Yolk production starts and ovulation happens 4 days later for B-eggs than for A-eggs (Grau 1982) and, for both A- and B-eggs, albumen deposition takes place when females are in the colony.

Because the amount of energy available for reproduction *via* food availability and/or female body reserves varies at several spatial and temporal scales, it is one of the most important factors underlying phenotypic plasticity in reproductive traits in oviparous animals (see reviews in Martin 1987; Du 2006). Penguins are capital breeders (Jönsson 1997; Meijer and Drent 1999). They acquire body reserves before and during migration to breeding sites and rely solely on these body reserves from their return to the colony to their first feeding trip, ca. 2–3 weeks after laying in female southern rockhopper penguins (Warham 1975). The quantity of energy females can invest in their clutch therefore depends on their own body reserve and we expect it to decrease as females fast in the colony (female body reserve depletion effect). Female body mass should therefore also be considered when examining egg mass variation.

We followed the return and egg laying of southern rockhopper penguins. We recorded female return dates, laying dates, and laying body masses, and collected their clutches to obtain yolk and albumen masses, in addition to total egg mass (used to define egg size). We predicted both A- and B-eggs to be heavier in females that returned to the colony early before laying relative to those that stayed longer at sea before and during yolk production. We also predicted egg masses, again for both A- and B-eggs, to increase with female body mass. Nevertheless, the migratory carry-over effect should be more visible in A-eggs and especially in their yolk as they are the first to be produced while females are at sea. Moreover, the female body reserve depletion effect should be more visible in albumen, which is entirely produced in the colony, and probably especially affects B-eggs, which are the last to be produced.

## Materials and Methods

### Study site and birds

The study was carried out at the “Settlement colony” (51°43′S, 61°17′W) on New Island, Falkland/Malvinas Islands between September and November 2009 and 2010. All applicable institutional and/or national guidelines for the care and use of animals were followed. The study was performed according to Belgian and Flemish law and was approved by the ethical committee on animal experimentation (ECD, ID number: 2011/44). All work was conducted under research licenses granted by the Environmental Planning Department of the Falkland Islands Government (research license numbers: R06/2009 and R15.2013).

**Figure 1:**
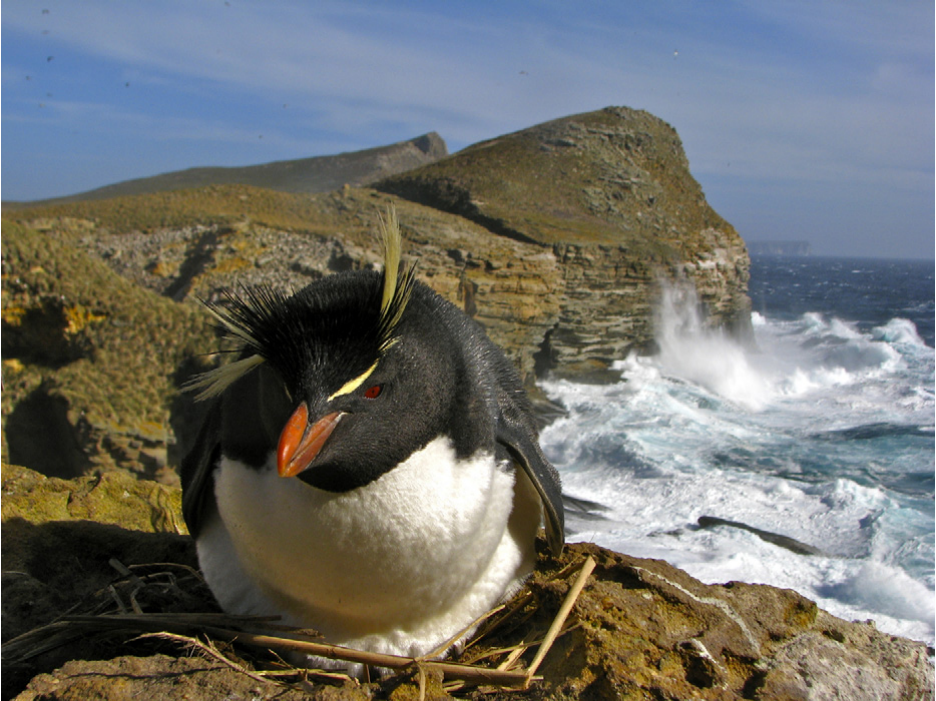
Southern rockhopper penguin *Eudyptes chrysocome*.

In 2010, this colony held about 7500 breeding pairs of southern rockhopper penguins (Fig.1). Birds mainly breed in open rocky areas fringed by tussock grass *Poa flabellata*. The breeding biology at this large colony has been described previously in Poisbleau et al. (2008). Briefly, males return to the colony first (early October) and establish nest sites. Females arrive a few days later, for pairing and copulation. Laying (late October/early November) and hatching (early December) intervals are relatively fixed; the second egg (B-egg) is generally laid 4 days after the first one (A-egg), incubation starts at clutch completion, but the A-egg usually hatches 1 day after the B-egg (reversed hatching asynchrony; St. Clair 1996).

### Adult survey

Since 2006, we have marked and followed around 450 females in the colony. They are equipped with 23-mm glass-encapsulated electronic transponders (TIRIS, Texas Instruments, Dallas, TX, USA) implanted under the skin of the back between the scapulae. A gateway system was set up in September 2009 and 2010, that is, before the return of the first adults to the breeding colony. It recorded the transponder number of each passing penguin equipped with a transponder as well as the date and the time of this passage. Positioned on a rock ledge that forms the only pathway for penguins breeding in the study colony, the gateway therefore recorded the individual return dates to the colony after the winter period.

After the return of the first females, we visited the colony daily to follow the egg laying of females equipped with a transponder. For the present study, we selected 75 nests (35 in 2009 and 40 in 2010) for which we obtained both the return date to the colony and the clutch initiation date of the females. We thus calculated the time interval (in days) between colony return and clutch initiation. We captured and weighed these females (to the nearest 20 g) on the day they laid their A-egg.

### Egg survey

The entire clutches of these 75 study nests (i.e., 75 A-eggs and 75 B-eggs) were collected for the present study as well as for the purpose of other studies involving compositional analyses (see Poisbleau et al. 2011a,b,c). In this context, and to avoid affecting breeding success, we simultaneously replaced these eggs with eggs found outside their own nests that we considered as recently lost by their original parents. Every egg was collected on the day it was laid. B-eggs were laid and collected three to 5 days after their sibling A-egg. We weighed them to the nearest 0.1 g using a digital balance. As incubation in rockhopper penguins typically does not start before clutch completion (Williams 1995), the A-eggs were not incubated at all and the B-eggs were not incubated for longer than 24 h at collection. We therefore assumed that embryo development and (potential) change in egg mass had not yet begun. No embryo development was observed during the preparation of any of the collected eggs. After collection, we froze the whole eggs at −20°C.

The same method was used to prepare all eggs (Poisbleau et al. 2009, 2011a,b,c). Briefly, we first removed the shell, while the egg was still frozen. Then, we separated the yolk from the albumen by taking advantage of the fact that albumen thaws more quickly than yolk. We recorded the mass of the yolk and albumen to the nearest 0.1 g using a digital balance. In addition to A-egg mass (in g) and B-egg mass (in g), we calculated the entire clutch mass (in g) as the sum of A-egg mass and B-egg mass and the intraclutch egg mass dimorphism as the difference in masses between A- and B-eggs. These four egg mass parameters were obtained not only for the total egg, but also for the egg components (yolk and albumen).

### Statistical analysis

Statistical analyses were conducted in IBM SPSS Statistics 20 for Windows (Chicago, IL, USA). Values are presented as means ± standard deviations (SD). We used dates as the number of days since the first of January of each year (Julian date) in order to standardize dates between breeding seasons. All date, time interval, female body mass, and egg mass parameters followed normal distributions (Kolmogorov–Smirnov tests, all *P *>* *0.05).

To investigate the relationships between variables, we used generalized linear mixed model procedures (GLMMs). We could not statistically examine the relationship between return date and the time interval between colony return and clutch initiation as the second variable has been calculated from the first one. We therefore focussed on the analyses with time interval between colony return and clutch initiation for the main analyses in this manuscript, but present additional analyses based on return date in the Appendix. GLMMs were run with female identity as subject and random factor and breeding season as repeated measure to control for the high repeatability in egg mass within individuals (Ojanen 1983; Christians 2002; Williams et al. 2009). The fixed factors and covariates introduced into each procedure are explained directly within the results and captions. The interactions between the fixed factor and the covariates were tested and removed from the models when not significant (*P *>* *0.05). We followed Nakagawa and Schielzeth (2013) to calculate marginal *R*^2^ values (for the variance explained only by fixed effects) and conditional *R*^2^ values (based on the variance explained by both fixed and random effects) for the identical models in the free software R (version 3.1.1.; R Core Team 2014). The parameter estimates *B* are given to describe the direction and magnitude of the relationships.

We additionally compared the coefficients of variation (CV) between A- and B-eggs using Levene’s test based on the median (Brown and Forsythe 1974; Schultz 1985).

## Results

### Return and laying patterns

In 2009 and 2010, female southern rockhopper penguins returned to the breeding colony between the 8th and the 18th of October (median date = 12th of October; Table1), with no difference between breeding seasons (GLMM with only breeding season as fixed factor: *F*_1,73_ = 1.209, *P *=* *0.275). Penguins initiated their clutches between the 25th of October and the 4th of November (median date = 31st of October; Table1), again with no significant difference between breeding seasons (GLMM with only breeding season as fixed factor: *F*_1,73_ = 0.152, *P *=* *0.698). The interval between colony return and clutch initiation was 18.44 ± 2.01 days (min–max: 15–24, *n *=* *75 females) and also did not differ between breeding seasons (GLMM with only breeding season as fixed factor: *F*_1,73_ = 2.590, *P *=* *0.112). Female body mass differed significantly between breeding seasons (GLMM with only breeding season as fixed factor: *F*_1,73_ = 10.354, *P *=* *0.002), with females being heavier in 2010 compared to 2009 (Table1). Female body mass was not related to return date, capture (i.e., clutch initiation) date, or to the interval between these dates (GLMMs with date or interval as fixed covariates: all *F*_1,73_ < 0.832, *P *>* *0.365).

**Table 1:** Return date and body mass at laying of females and yolk mass, albumen mass, shell mass, and total egg mass by breeding season for A- and B-eggs separately. Means ± standard deviations (in days) for dates (dd/mm) and means ± standard deviations for masses in g. *N* = 35 females, A- and B-eggs in 2009. *N* = 40 females, A- and B-eggs in 2010

	2009		2010	
	A-eggs	B-eggs	A-eggs	B-eggs
Female return date	12/10 ± 2.5 day		11/10 ± 1.8 day	
Female laying mass	3103 ± 162		3187 ± 183	
Laying date	30/10 ± 2.0 day	03/11 ± 2.0 day	30/10 ± 1.9 day	03/11 ± 2.0 day
Yolk mass	19.33 ± 2.03	22.18 ± 2.08	19.49 ± 2.39	21.83 ± 2.33
Albumen mass	63.57 ± 7.75	80.51 ± 6.76	66.05 ± 7.11	82.51 ± 7.09
Shell mass	13.46 ± 1.26	16.91 ± 1.50	12.34 ± 1.19	14.97 ± 1.32
Total egg mass	96.36 ± 9.46	119.6 ± 8.1	97.87 ± 9.40	119.3 ± 9.4

**Figure 2:**
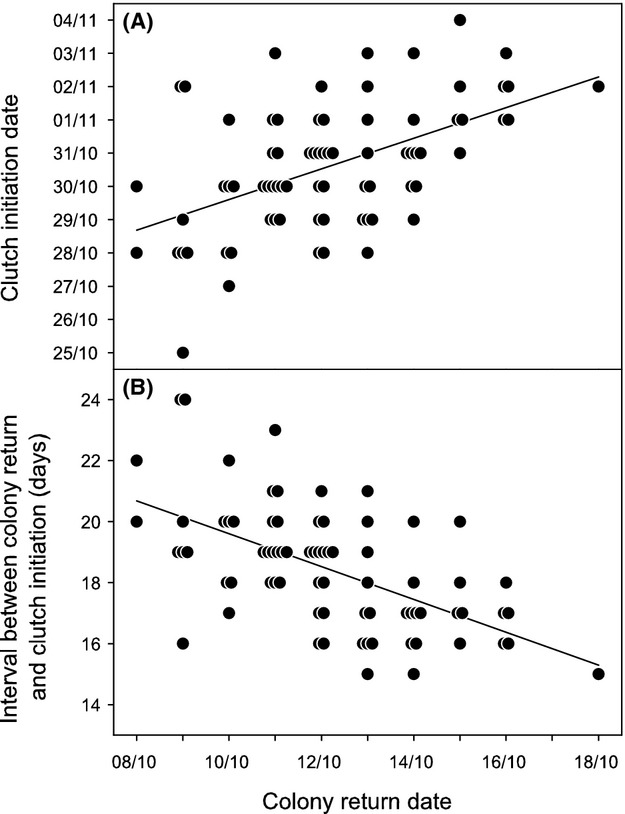
(A) Individual female clutch initiation (i.e., A-egg laying) date and (B) time interval (in days) between colony return and clutch initiation according to the date of return to the colony after winter in southern rockhopper penguins *Eudyptes chrysocome*. Lines are linear regressions and identical points have been spread out slightly for clarity. *n *=* *75 females.

Return date determined clutch initiation date (GLMM with only return date as covariate: *F*_1,73_ = 36.472, *P *<* *0.001). Females that returned late also laid late (*B *=* *0.461; Fig.2A) even though they shortened the interval between colony return and clutch initiation compared to females that returned early (Fig.2B).

### Variation in egg mass

Yolk mass, albumen mass, and total egg mass were more variable for A-eggs than for B-eggs, with significant differences in the coefficients of variation (CVs) for albumen mass and total egg mass (Table2).

**Table 2:** Means ± standard deviations (SD) and coefficients of variations (CV) for yolk mass (in g), albumen mass (in g), and total egg mass (in g) of A- and B-eggs. *n *=* *75 clutches. Comparisons between A- and B-eggs use Levene’s test based on the median. Significant *P*-values, *P *<* *0.05, are marked in bold

	A-eggs	B-eggs	*F*_1,148_	*P*
Yolk mass				
Mean ± SD	19.42 ± 2.22	22.00 ± 2.21		
CV	11.41%	10.04%	1.349	0.247
Albumen mass				
Mean ± SD	64.90 ± 7.47	81.58 ± 6.96		
CV	**11.51%**	**8.54%**	**5.590**	**0.019**
Total egg mass				
Mean ± SD	97.17 ± 9.37	119.44 ± 8.76		
CV	**9.67%**	**7.33%**	**5.439**	**0.021**

**Figure 3:**
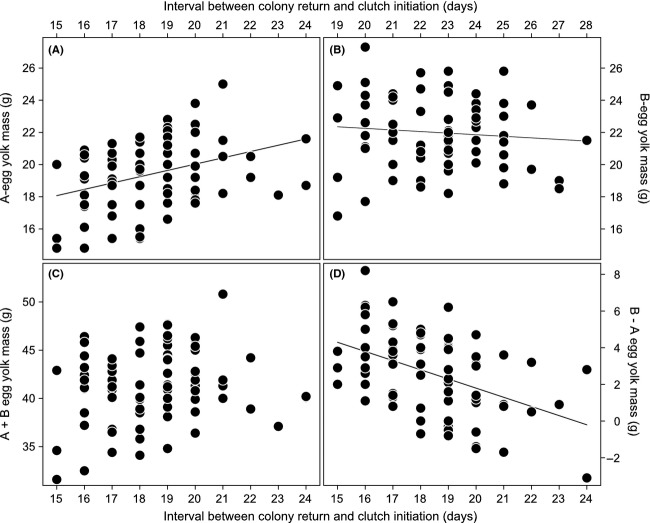
Relationship between (A) A-egg yolk mass (in g), (B) B-egg yolk mass (in g), (C) entire clutch yolk mass (in g), and (D) intraclutch yolk mass dimorphism (B-egg–A-egg) and the time interval (in days) between colony return and clutch initiation. Regression lines are shown where *P *<* *0.05. *n *=* *75 clutches.

**Figure 4:**
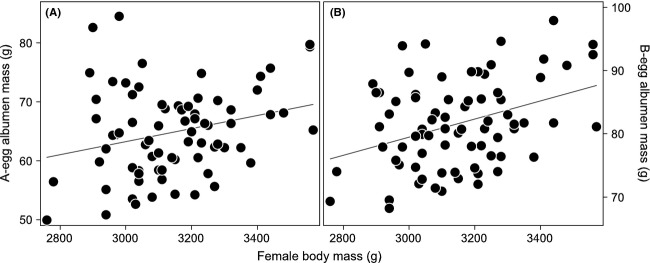
Relationship between (A) A-egg albumen mass (in g), (B) B-egg albumen mass (in g), and female body mass (in g) on the day they laid their A-egg. Regression lines are shown where *P *<* *0.05. *n *=* *75 clutches.

Consistent with our expectation based on the carry-over effect hypothesis, A-egg yolk mass was correlated with the interval between colony return and clutch initiation (Table3a): females that spent a long time in the colony before laying produced heavier A-egg yolks than females that spent a short time in the colony (Fig.3A). B-egg yolk mass also varied with this interval (Table3b) but in the opposite direction: females that spent a shorter time in the colony produced heavier B-egg yolks (Fig.3B). The interval between colony return and clutch initiation had a positive effect on A-egg albumen mass, while this was not the case for the B-egg albumen mass (Table3b). Female body mass had no significant influence on yolk mass in either A- or B-eggs (Table3a and b) but, as expected, albumen mass increased with female body mass for both A- and B-eggs (Table3a and b; Fig.4A and B). Total egg mass therefore increased with the interval between colony return and clutch initiation and with female body mass for A-eggs (Table3a) but only with female body mass for B-eggs (Table3b).

**Table 3:** Results of the generalized linear mixed model procedures (GLMMs) on yolk mass, albumen mass, and total egg mass (in g, dependent variables) for (a) A-eggs, (b) B-eggs, (c) entire clutches, and (d) intraclutch egg mass dimorphism (difference between B- and A-eggs) of southern rockhopper penguins *Eudyptes chrysocome*. GLMMs were run with female identity as subject and random factor and breeding season (2009 or 2010) as repeated measure. Breeding season was included as a fixed factor. Female body mass (in g) and the time interval (in days) between colony return and clutch initiation were included as covariates. *n *=* *75 clutches. The interactions between the fixed factor and the covariates were tested and removed from the models when not significant (*P *>* *0.05). Significant *P*-values, *P *<* *0.05, are marked in bold. The parameter estimates *B* are given to describe the direction and magnitude of the relationships. 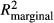 values represent the variance explained only by fixed effects and 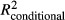 the variance explained by both fixed and random effects

	Yolk mass			Albumen mass			Total egg mass		
	*F*_1,71_	*P*	*B*	*F*_1,71_	*P*	*B*	*F*_1,71_	*P*	*B*
(a) A-egg	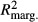 = 0.159			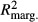 = 0.091			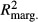 = 0.092		
	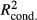 = 0.637			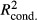 = 0.905			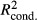 = 0.918		
Breeding season	1.630	0.206	−0.487	2.478	0.120	1.188	0.404	0.527	−0.553
Female mass	2.562	0.114	0.002	**4.312**	**0.041**	**0.008**	**5.401**	**0.023**	**0.010**
Interval	**11.02**	**0.001**	**0.380**	**5.581**	**0.021**	**0.613**	**10.32**	**0.002**	**0.973**
(b) B-egg	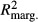 = 0.044			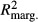 = 0.164			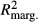 = 0.125		
	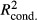 = 0.729			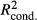 = 0.768			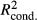 = 0.785		
Breeding season	0.583	0.448	−0.280	0.912	0.343	1.017	0.709	0.403	−1.112
Female mass	0.061	0.806	<0.001	**12.28**	**0.001**	**0.016**	**10.88**	**0.002**	**0.019**
Interval	**3.958**	**0.050**	−**0.233**	0.072	0.789	0.092	0.416	0.521	−0.276
(c) Clutch	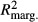 = 0.024			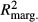 = 0.131			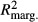 = 0.095		
	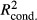 = 0.749			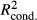 = 0.874			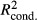 = 0.894		
Breeding season	1.554	0.217	−0.741	2.023	0.159	2.307	0.531	0.469	**−**1.361
Female mass	0.682	0.412	0.002	**10.20**	**0.002**	**0.025**	**10.56**	**0.002**	**0.030**
Interval	0.358	0.551	0.114	1.058	0.307	0.565	0.445	0.507	0.428
(d) Dimorphism	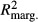 = 0.239			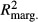 = 0.160			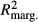 = 0.234		
	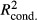 = 0.428			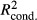 = 0.516			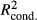 = 0.543		
Breeding season	0.639	0.427	−0.898	0.008	0.930	−0.039	0.018	0.895	−0.110
Female mass	1.288	0.260	0.004	0.880	0.351	−0.001	2.866	0.095	0.005
Interval	**21.26**	**<0.001**	−**1.449**	**22.20**	**<0.001**	−**0.525**	**11.55**	**0.001**	−**0.799**

Notably, the three fixed effects (breeding season, female body mass, and time interval) explained between 4% and 16% of variance in egg masses. With 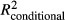 values of up to 92%, all these models reflected a high proportion of explained variance, which was, however, largely ascribed to female identity and not to breeding season, female body mass, or time interval (Table3).

### Entire clutch masses and intraclutch dimorphism in mass

Entire clutch yolk mass, albumen mass, and total egg mass did not vary with the time females spent in the colony before laying (Table3c; Fig.3C) but entire clutch albumen mass and total egg mass increased with female body mass (Table3c). These effects were also reflected by the higher explanatory power of models for albumen and total egg mass compared to yolk mass (see both 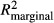 and 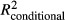 values; Table3c).

In contrast, intraclutch dimorphism in yolk mass, albumen mass, and total egg mass did not vary with female body mass (Table3d) but did vary with the time females spent in the colony before laying (Table3d). Females laid less dimorphic clutches when they spent more time in the colony before laying (Table3d; Fig.3D). Therefore, the fixed effects (breeding season, female body mass, and time interval) together explained up to 24% of variance in the models for intraclutch dimorphism, while the birds’ identity (as random factor) explained less than in the previous models on A-eggs, B-eggs, and entire clutches (cf. 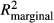 and 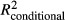 values; Table3d).

## Discussion

The foremost aim of this study was to investigate whether migration and reproduction constraints may act on phenotypic plasticity in egg laying behavior, specifically on the intraclutch egg size dimorphism (including the different egg components) in *Eudyptes* penguins. Alongside predictions based on the migratory carry-over effect, we also analyzed the influence of female body mass on the different egg components and both are discussed subsequently, after briefly viewing some more general results on return and laying patterns.

### Return and laying patterns

In line with previous observations (Strange 1982), return date, clutch initiation date, and the consequent time interval between these two dates did not differ between the two breeding seasons of this study. Unsurprisingly, clutch initiation date was determined by return date, even though females that returned late shortened the interval between their return and clutch initiation. Both effects have been described before in birds (Hupp et al. 2006), including penguins (Emmerson et al. 2011) and especially southern rockhopper penguins (Poisbleau et al. 2013). These results indicate that the time interval between return and clutch initiation is a function of return date and suggest that both return date and time interval might be important as regards female investment into clutches. As these two parameters are not independent of each other, it was not possible to include them within the same model. We therefore performed similar analyses with return date (instead of time interval; see Appendix). These additional results show that return date explains less variation in the egg masses than time interval, and we here consequently focus on the results related to time interval.

In contrast to other studies on terrestrial species in which female body mass or condition is often related to the timing of breeding (see, e.g., Drent and Daan 1980; Bêty et al. 2003), individual female body mass at laying was not related to the return date, clutch initiation date, or the interval between these two dates. Nonetheless, females were heavier in 2010 compared to 2009, and we therefore consistently included breeding season as a factor in the statistical models. The interactions between breeding season and other variables were also examined, but none was significant. We observed that A-eggs were more variable in mass than B-eggs. This may be linked to the fact that B-egg formation takes place almost entirely on land, while a substantial part of the A-egg formation takes place at sea, a contrasting energetic environment. Indeed, if we assume that egg production lasts for 23 days (as per Grau 1982), females spent 4.59 ± 1.94 days (min–max: 0–8) at sea during the production of the A-eggs and only 1.03 ± 1.29 days (min–max: 0–4, *n *=* *75 females) at sea during the production of the B-eggs. This also fits with the literature (Ancel et al. 2013). We therefore note that 93.3% of A-eggs (i.e., 73) started to be produced at sea, but only 68.0% of B-eggs (i.e., 51). In other words, 32.0% of B-eggs may have been entirely produced in the colony versus only 2.7% of A-eggs.

### Migratory carry-over effects

Carry-over effects are widespread, driven by multiple factors, and could be responsible for much of the observed variation in performance among individuals (Harrison et al. 2011). According to their recent definition applied to ecological and evolutionary studies, carry-over effects occur in any situation in which an individual’s previous history and experience explains their current performance in a given situation (O’Connor et al. 2014). Here, we followed the hypothesis of Crossin et al. (2010) to test whether any prebreeding migratory effect may influence breeding performances and more specifically whether the degree of overlap between migratory activities and yolk production experienced by individual females (or equivalently, the interval between colony return and clutch initiation) influences female investment in egg mass in southern rockhopper penguins. We hypothesized that a difference in the migratory carry-over effect between A- and B-eggs could explain the puzzling intraclutch egg size dimorphism observed in *Eudyptes* penguins.

Indeed, females laid heavier A-eggs when they spent more time in the colony before laying. This effect was consistent for all three A-egg components (yolk mass, albumen mass, and total egg mass) and supports the carry-over effects on A-eggs. However, while we expected the carry-over effect to be smaller for B-eggs than for A-eggs, we could not confirm any carry-over effect in B-eggs or their components. The interval between colony return and clutch initiation was negatively correlated with B-egg yolk mass and did not correlate with the other egg components. This result may reflect the fact that B-eggs were barely formed at sea and could in addition suggest that the longer females fast in the colony before laying, the less resources they can devote to egg production.

### Female body mass effects

While penguins that return early and spend more time in the colony might reduce the conflict between migration and reproduction, they also face a longer fasting period. During the first few days of fasting, individuals use stomach and gut contents and extracellular fluids and a rapid decrease in mass is generally observed in mammals and birds, including penguins (see, e.g., Runcie and Hilditch 1974; Cherel et al. 1993). Afterward, there is a slower linear decline in body mass caused largely by the loss of water and lipids (Groscolas 1988; Cherel et al. 1993).

In the present study, yolk production began before fasting and continued to the middle of the fasting period, whereas albumen deposition occurred when females had already fasted for 14–23 days. Females must therefore rely more on their own body reserves during albumen deposition than during yolk production. This might explain the lack of association between female body mass and yolk mass alongside the expected positive association between female body mass and albumen mass observed in both eggs and for the whole clutch. The positive correlation we found between female body mass and albumen mass (and total egg mass) is in line with other studies (e.g., Drent and Daan 1980; Hepp et al. 1987; Bêty et al. 2003; Figuerola and Green 2006; Stein and Williams 2013). Moreover, in the present study, we recorded female body mass on the day females laid their A-egg, that is, exactly between A-egg and B-egg albumen deposition, which might have further enhanced the effect.

### Implications for intraclutch egg size dimorphism

In summary, our results suggest that the constraints that act on reproductive traits differ between individual eggs within clutches and between egg components within eggs. In other words, the eggs and their components are not equally sensitive to the migratory carry-over and female body mass effects. These different constraints are directly linked to the timing of production of these eggs and their components. Migratory constraints had a stronger effect on early-formed components (A-egg yolk), whereas female body reserves influenced the later-formed components (B-egg albumen) to a larger extent. This resulted in an increase in A-egg, B-egg, and entire clutch masses as female body mass increased and a decrease in the intraclutch egg size dimorphism as the interval between colony return and clutch initiation increased. Conversely, intraclutch egg size dimorphism was not influenced by female body mass, neither was entire clutch mass affected by the time interval. The increase in egg/clutch masses with female body mass is common in birds and other taxa (Honěk 1993; Wendeln 1997; Ellis et al. 2000; Paitz et al. 2007). The link between intraclutch egg size dimorphism – which was also relatively less dependent on female identity than egg and clutch masses were – and migratory constraint deserves future investigations.

An especially exciting part of the present results is that rockhopper penguins might generate plasticity in intraclutch egg size dimorphism by something they can control: the duration/timing of foraging prior to the breeding season or, more proximately, the foraging locations. Thus, birds that forage long may be more likely to return late (forcing a shorter interval between return and laying) and to compensate for that time constraint with reduced investment in A-eggs compared to birds that forage short and return earlier. Low investment in A-eggs might also be a way to maintain high body reserves for the chick-rearing period. The fact that B-egg mass did not vary with time interval between colony return and clutch initiation (or return date; see Appendix) suggests that females maintain a stable investment strategy for their second egg while enhancing the likelihood of brood reduction under certain conditions. Our results may therefore shed new light on the mechanisms that cause the maintenance of the potentially maladaptive two-egg clutches in *Eudyptes* penguins (Stein and Williams 2013) and question whether other penguin species that forage further away from their colonies in winter might have evolved one-egg clutches because of their sensitivity to the migratory carry-over effect. If this is the case, one could expect the largest intraclutch egg size dimorphism in those *Eudyptes* penguin species with the farthest winter dispersal. Potentially, the effect of the migratory carry-over effect might even be so severe as to limit the viability of A-eggs in some *Eudyptes* penguins and thus support their intentional ejection by their parents (St. Clair 1995). In these species, the two-egg clutch would clearly reflect a maladaptation, while this may not be the case in some other *Eudyptes* species (including our study species), in which A-eggs may still have an insurance value, as hatching success appeared to be independent of their overall size (St. Clair et al. 1995; St. Clair and St. Clair 1996). We, however, do not know how the reduced yolk mass may affect this insurance value. Ideally, the relationship between prebreeding migratory behavior (distance to colony and length of time to return to the colony) and clutch size dimorphism could be investigated in a multispecies study in several *Eudyptes* penguins. Ultraminiaturized electronic devices such as miniaturized global locating system units (GLS) that now allow bird movements (distance and time) to be followed over a long time could enable such a study.

Moreover, in order to further improve our understanding of the relative effects of such migratory constraints and female body mass on reproduction, we also advise controlling for certain other confounding effects. As such, the fact that egg production occurs in two different environments, with the option to forage at sea while facing migratory constraints, yet to fast while on land (without the migratory constraint) complicates analyses and interpretation. Furthermore, food availability (affecting female body mass and potentially breeding behavior; Le Maho et al. 1993) and temperatures (that may affect thermoregulatory costs both on land and at sea; Luna-Jorquera and Culik 2000; Schmidt et al. 2006) may affect egg composition (Ardia et al. 2006; Cucco et al. 2009; Saino et al. 2010).

## Appendix

Results of the generalized linear mixed model procedures (GLMMs) on yolk mass, albumen mass, and total egg mass (in g, dependent variables) for (a) A-eggs, (b) B-eggs, (c) entire clutches, and (d) intra-clutch egg mass dimorphism (difference between B- and A-eggs) of southern rockhopper penguins *Eudyptes chrysocome*. GLMMs were run with female identity as subject and random factor and breeding season (2009 or 2010) as repeated measure. Breeding season was included as a fixed factor. Female body mass (in g) and the date that the female arrived in the colony (Julian date) were included as covariates. *n *=* *75 clutches. The interactions between the fixed factor and the covariates were tested and removed from the models when not significant (*P *>* *0.05). Significant *P*-values, *P *<* *0.05, are marked in bold. The parameter estimates *B* are given to describe the direction and magnitude of the relationships.  values represent the variance explained only by fixed effects and  the variance explained by both fixed and random effects.

**Table:**

	Yolk mass			Albumen mass			Total egg mass		
	*F*_1,71_	*P*	*B*	*F*_1,71_	*P*	*B*	*F*_1,71_	*P*	*B*
(a) A-egg	= 0.076			= 0.060			= 0.044		
	= 0.574			= 0.914			= 0.910		
Breeding season	0.686	0.410	−0.329	**5.574**	**0.021**	**1.718**	0.034	0.855	0.167
Female mass	3.051	0.085	0.003	**4.350**	**0.041**	**0.008**	**5.317**	**0.024**	**0.024**
Return date	**4.297**	**0.042**	**−0.202**	0.223	0.638	0.090	0.052	0.821	0.821
(b) B-egg	= 0.013			= 0.165			= 0.125		
	= 0.640			= 0.762			= 0.762		
Breeding season	1.271	0.263	−0.444	0.955	0.332	1.026	0.993	0.322	−1.327
Female mass	0.106	0.746	0.001	**12.34**	**0.001**	**0.016**	**10.64**	**0.002**	**0.019**
Return date	0.109	0.742	0.033	0.160	0.690	**−**0.109	0.001	0.974	0.011
(c) Clutch	= 0.022			= 0.120			= 0.089		
	= 0.748			= 0.889			= 0.905		
Breeding season	1.534	0.220	−0.723	3.374	0.070	2.805	0.276	0.601	−0.922
Female mass	0.741	0.392	0.002	**10.50**	**0.002**	**0.025**	**10.80**	**0.002**	**0.030**
Return date	0.897	0.347	−0.138	0.080	0.778	0.114	0.168	0.683	0.190
(d) Dimorphism	= 0.069			= 0.034			= 0.028		
	= 0.195			= 0.462			= 0.396		
Breeding season	0.234	0.630	−0.242	0.434	0.512	−0.578	1.698	0.197	−1.664
Female mass	1.420	0.237	0.002	2.100	0.152	0.004	0.581	0.448	0.003
Return date	2.617	0.110	0.189	0.001	0.984	0.004	0.543	0.464	0.230
